# Brugada Syndrome Unmasked by Use of Testosterone in a Transgender Male: Gender Trumps Sex as a Risk Factor

**DOI:** 10.19102/icrm.2019.100202

**Published:** 2019-02-15

**Authors:** Tina C. Sichrovsky, Suneet Mittal

**Affiliations:** ^1^The Snyder Center for Comprehensive Atrial Fibrillation, Valley Health System, Ridgewood, NJ, USA

**Keywords:** Brugada syndrome, testosterone, transgender

## Abstract

We describe a genetic female living as a transgender male through the use of exogenous testosterone supplementation. He developed Brugada pattern (that was unrecognized) and subsequently had an out-of-hospital cardiac arrest. Sustained ventricular arrhythmias were suppressed through treatment with quinidine; however, this medication could only be administered at very low doses due to the development of angioedema at higher doses. Subsequently, the patient required endocardial ablation for elimination of highly symptomatic, repetitive monomorphic ventricular ectopy. This case highlights the presentation of a unique patient in whom a channelopathy was unmasked by the patient’s lifestyle, suggesting that gender trumps sex when it comes to arrhythmia risk in patients at risk for Brugada syndrome.

A 63-year-old Caucasian male presented with cardiac arrest. At the time of our initial encounter with the patient, he was undergoing therapeutic hypothermia in the coronary care unit. The patient’s wife reported that he had a history of paroxysmal atrial fibrillation and night terrors. The patient apparently awoke many times in the middle of night screaming that he was “going to die.” A prior sleep study had been unrevealing.

The patient had insisted that his wife learn cardiopulmonary resuscitation (CPR). Remarkably enough, on the night of admission, the patient’s wife had had to perform CPR on her husband when she found him to have stopped breathing. He had regained consciousness by the time paramedics arrived; the patient refused to go to the hospital for evaluation. He went back to sleep, but shortly thereafter, he arrested a second time and, this time, the wife could not resuscitate him. Emergency medical services personnel were called, and the patient underwent external shock twice via an automated external defibrillator for polymorphic ventricular tachycardia (VT).

The patient was admitted to the hospital and therapeutic hypothermia was initiated. An electrocardiogram (ECG) obtained while the patient was hypothermic showed sinus rhythm; additionally, there were occasional premature ventricular contractions with left bundle branch block, inferior axis morphology **(Figure 1)**. Five months before the cardiac arrest, the patient had undergone an ECG recording taken as part of his evaluation for atrial fibrillation **(Figure 2)**. The ECG was consistent with type I Brugada pattern. However, this was not recognized by his physician; rather, the patient underwent an unremarkable evaluation for ischemia, given the ST-segment elevations.

The patient reportedly had no family history of cardiac arrest or Brugada syndrome and ultimately made a complete neurologic recovery. It was revealed at this point that the patient is genetically female. However, with the use of testosterone (Depo^®^-Testosterone 200 mg intramuscularly twice weekly; Pfizer, New York, NY, USA) for about 20 years, he was living the life of a transgender male. He had no other medical history (including prior syncope) and was taking no other medications.

He received a dual-chamber implantable cardioverter-defibrillator (ICD) (an atrial lead was implanted to permit atrial pacing for managing severe nocturnal bradycardia) and was discharged home. Four weeks later, he received several ICD shocks for ventricular fibrillation (VF) storm while asleep. He was admitted and quinidine sulfate was initiated. This was changed to quinidine gluconate ER 324 mg due to the unavailability of the former. Subsequently, after a few days, he developed angioedema every time he took quinidine.

He was advised to stop the testosterone, but he did not consider this to be a viable option, as his identity was dependent upon its continued use. We referred the patient to an allergist. The patient was prescribed levocetrizine 5 mg at bedtime, which also allowed him to tolerate once-daily dosing of quinidine gluconate ER at bedtime.

During follow-up, the patient complained of frequent palpitations due to a high burden (7.7%) of repetitive monomorphic ventricular ectopy. The patient underwent an electrophysiology study; ectopy was found to originate from the right ventricular (RV) outflow tract and was successfully ablated. The patient has remained free of symptoms, ventricular ectopy, and sustained ventricular arrhythmias since. His ICD diagnostics have confirmed an absence of ventricular ectopy.

## Discussion

Brugada syndrome is an autosomal dominant inherited channelopathy with a phenotypic predilection for the male gender. In fact, Brugada syndrome appears to occur eight to 10 times more frequently in males despite being equally inherited by both genders^1^; there is also a higher incidence of spontaneous type I ECG Brugada pattern, fatal arrhythmias, and overall worse prognosis in males.^2^ The low penetrance of the disease in females led to the Southeast Asian custom of men dressing in women’s clothes at bedtime to fool the “evil spirits” that were believed to target males in their sleep.^1^ The sex-related difference in the phenotypic expression of Brugada syndrome is more pronounced than with any other autosomally transmitted arrhythmic syndrome.

Brugada syndrome is linked to mutations in *SCN5A*, the α subunit of the sodium channel, resulting in a loss of function.^3,4^ The genetic diversity of the syndrome remains to be more fully delineated, as *SCN5A* mutations account for only approximately 20% of Brugada cases.^5^ Accentuation of the epicardial action potential notch and eventual loss of the epicardial action potential dome results in ST-segment elevation, phase II reentry, and polymorphic VT/VF.^6–8^ The proposed mechanism involves a rebalancing of the currents available at the end of phase I of the epicardial action potential. Diminution of inward currents (*I*_Na_ and *I*_Ca_) or enhancement of outward currents [*I*_to_, *I*_K-ATP_, *I*_Kr_, *I*_Ks_, *I*_Cl(Ca)_] can result in an accentuation of the epicardial action potential notch as well as all-or-none repolarization at the end of phase I. The presence of a prominent *I*_to_-mediated action potential notch appears to be a prerequisite. Indeed, the presence of a much greater *I*_to_ in the RV versus the left ventricular (LV) epicardium accounts for the RV nature of the disease.^6,8^ In animal models, *I*_to_ current density of the RV epicardium and time constant for inactivation are significantly larger in males than in females, creating greater transmural dispersion of repolarization.^9^ This induces greater transmural and epicardial dispersion of repolarization in males, which facilitates the development of the Brugada-like ECG pattern and VT.^9^ The less prominent *I*_to_ in females is due in large part to the more rapid inactivation kinetics of the channel in females. Other animal data indicate that although *I*_to_ is smaller in the LV epicardium than in the RV epicardium, sex-related differences are not observed in the former.^9^

However, other factors like hormonal influences seem to be involved in the gender-related clinical differences. Certain ion channels (eg, *CACNA1C, SCN5A*) play a pathogenic role in Brugada syndrome and are particularly sensitive to testosterone. J-point elevation and ST elevation in healthy subjects are greater in men than in women, but only after puberty. The effects of androgens on the early phases of repolarization could be explained by their potential to increase net outward currents (mainly potassium), accentuate transmural dispersion of repolarization, and potentially cause loss of the epicardial dome of the action potential, leading to ST-segment elevation, facilitating arrhythmia susceptibility.^2^ The intergender differences are no longer evident under androgen-deprivation therapy for prostate cancer.^10^

Case reports of males treated with surgical or pharmacological castration have included the disappearance of Brugada ECG pattern.^10,11^ A Japanese study evaluated 21 prostate cancer patients undergoing androgen-depriving therapy in whom standard 12-lead ECGs before and after treatment were recorded. Three ST-segment levels were analyzed: the J-point level and the middle and end of the ST segment, respectively. Female and male controls were used for comparison. The study showed that androgen-deprivation therapy significantly lowered all three ST-segment parameters to resemble the ST levels of the age-matched control females.^10^

Our patient presents several challenges due to his dependence on testosterone treatment to protect his identity and quality of life as well as the existence of a severe allergy to quinidine, which is typically an effective antiarrhythmic drug in patients with Brugada syndrome and sustained ventricular arrhythmias.^12^ At the time of presentation, he was taking 200 mg of testosterone intramuscularly twice weekly, which is considered to be an average dose and is in line with the 50-mg to 400-mg dose administered every two weeks to four weeks in patients with male hypogonadism.

Interestingly, even though ventricular ectopy in Brugada syndrome usually originates from the epicardial RV flow tract,^13^ our patient’s ectopy could successfully be ablated from the endocardial aspect of the RV outflow tract. We are uncertain as to whether these ectopics triggered his episodes of sustained VF, as his ICD was not programmed to store intracardiac electrogram information at the onset of arrhythmia. Interestingly, the patient only had rare premature ventricular contractions (PVCs) during his hospitalization following the initial cardiac arrest and, subsequently, these PVCs persisted despite the suppression of sustained ventricular arrhythmias with low-dose quinidine.

In summary, we describe a genetic female living as a transgender male through the use of exogenous testosterone supplementation. He developed a Brugada pattern (that was unrecognized) and subsequently had an out-of-hospital cardiac arrest. Sustained ventricular arrhythmias were suppressed through treatment with quinidine; however, this could only be administered at very low doses due to the development of angioedema at higher ones. Subsequently, the patient required endocardial ablation for the elimination of highly symptomatic repetitive monomorphic ventricular ectopy. This case highlights a unique patient in whom a channelopathy was unmasked by the patient’s lifestyle, suggesting that gender trumps sex when it comes to arrhythmia risk in patients at risk for Brugada syndrome.

## Figures and Tables

**Figure 1:**
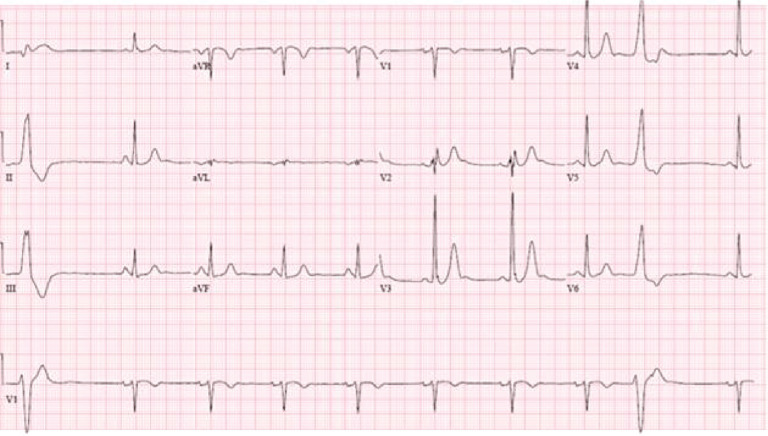
ECG obtained on the day of the patient’s cardiac arrest, while the patient was undergoing therapeutic hypothermia.

**Figure 2:**
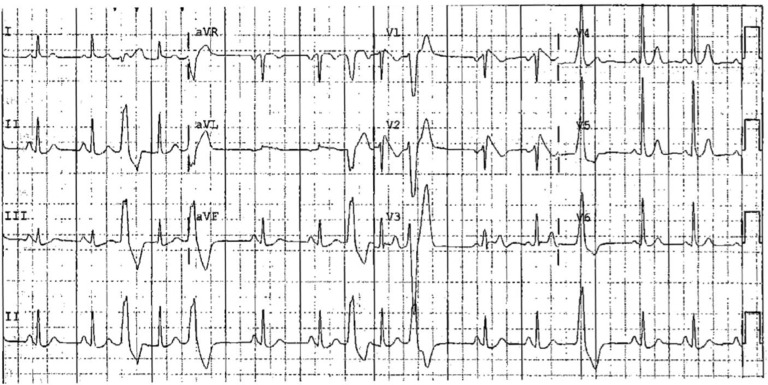
ECG obtained five months before the cardiac arrest. The presence of type I Brugada pattern was not recognized; rather, the patient underwent an unremarkable “ischemia evaluation” given the presence of ST-segment elevation in leads V1 and V2.
